# Carbon Footprints for Food of Animal Origin: What are the Most Preferable Criteria to Measure Animal Yields?

**DOI:** 10.3390/ani2020108

**Published:** 2012-03-27

**Authors:** Gerhard Flachowsky, Josef Kamphues

**Affiliations:** 1 Institute of Animal Nutrition, Friedrich-Loeffler-Institute (FLI), Federal Research Institute for Animal Health, Bundesallee 50, 38116 Braunschweig, Germany; 2 Institute of Animal Nutrition, University of Veterinary Medicine Hannover, Foundation, Bischofsholer Damm 15, 30173 Hannover, Germany; E-Mail: Josef.Kamphues@tiho-hannover.de

**Keywords:** food of animal origin, carbon footprints, system boundaries, milk, eggs, carcass, meat, edible protein

## Abstract

**Simple Summary:**

Greenhouse gas emissions from animal production are substantial contributors to global emissions. Therefore Carbon Footprints (CF) were introduced to compare emissions from various foods of animal origin. The CF for food of animal origin depends on a number of influencing factors such as animal species, type of production, feeding of animals, level of animal performance, system boundaries and output/endpoints of production. Milk and egg yields are more clearly defined animal outputs of production than food from slaughtered animals. Body weight gain, carcass weight gain, meat, edible fractions of carcass or edible protein are measurable outputs of slaughtered animals. The pros and contras of various outcomes under special consideration of edible protein are discussed in this paper.

**Abstract:**

There are increasing efforts to determine the origin of greenhouse gas emissions caused by human activities (including food consumption) and to identify, apply and exploit reduction potentials. Low emissions are generally the result of increased efficiency in resource utilization. Considering climate related factors, the emissions of carbon dioxide, methane and laughing gas are summarized to so-called carbon footprints (CF). The CF for food of animal origin such as milk, eggs, meat and fish depend on a number of influencing factors such as animal species, type of production, feeding of animals, animal performance, system boundaries and outputs of production. Milk and egg yields are more clearly defined animal yields or outcomes of production than food from the carcasses of animals. Possible endpoints of growing/slaughter animals are body weight gain, carcass weight gain (warm or cold), meat, edible fractions or edible protein. The production of edible protein of animal origin may be considered as one of the main objectives of animal husbandry in many countries. On the other hand, the efficiency of various lines of production and the CF per product can also be easily compared on the basis of edible protein. The pros and contras of various outputs of animal production under special consideration of edible protein are discussed in the paper.

## 1. Introduction

The current world situation is characterized by a growing population and a higher need for feed and food. These facts are, in turn, associated with a growing demand for limited natural resources such as fuel, land area, water, *etc.*, and with elevated emissions with greenhouse gas (GHG) potential. Such gases can include, e.g., carbon dioxide (CO_2_), methane (CH_4_), laughing gas (N_2_O) and other substances (e.g., N, P, trace elements, *etc.*; (e.g., [1,2,3,4,5]). During the last few years special attention has been devoted to various gases because of their greenhouse gas potential. This increase is discussed in the context of global warming and possible climate change [2].

Agriculture and especially animal husbandry are considered as important greenhouse gas sources because of the high greenhouse potential of their emissions (e.g., CO_2_ × 1; CH_4_ × 23 and N_2_O × 296; [2]). So-called Carbon Footprints (CF; Life cycle assessments (LCA), Eco-Balances) consider the greenhouse gas potential of climate relevant gases and are expressed relative to one gram or kilogram of CO_2_ equivalent (CO_2eq_) per product unit. 

Various authors calculated such CF for agriculture in general, but also for separate segments. For example, according to Steinfeld *et al.* [1] livestock production contributes about 18% to the global anthropogenic GHG emissions. O`Mara [6] has reported that animal agriculture is responsible for 8–10.8% of the global GHG emissions. After Lesschen *et al.* [7] livestock farming contributes to global warming with about 10% of total GHG emissions from the EU-27. FAO [4] asserts that the global dairy sector contributes with 3.0 to 5.1% to total anthropogenic greenhouse gases, but Sevenster and Jones [8] calculated only 1.2% from dairy livestock to the global greenhouse gas emissions. The highest variation of global GHG emissions from livestock is mentioned by Herrero *et al.* [9] with a range from 8 to 51%. Detailed information about the GHG emission in the EU is given by Leip *et al.* [10]. From a total of about 660 million tonnes CO_2eq_ per year from livestock, about 65% come from ruminants (production of milk, beef, sheep and goats [10]). Methodical and regional differences make it difficult to compare such values, to make conclusions or to give data based advice to policy makers. The objectives of CF are to sensitize producers and consumers for an efficient use of fossil carbon sources and to reduce greenhouse gas emissions per unit of product.

During the last 10 years many studies dealt with calculations of CF for almost all products resulting from human activities, including production of food of animal origin (e.g., [4,5,7,10,11,12,13,14,15,16,17]). The large range in CF when comparing results of various authors depends on many influencing factors as exemplary shown in Table 1 and Table 2 for milk and beef. The CF for milk varies between 0.4 and 1.5 kg CO_2eq_/kg milk, taking different world regions into account, between 1.3 (Europe and North America) and 7.5 kg CO_2eq_/kg in sub-Saharan Africa [4] (see Table 1). Furthermore, most authors considered only the emissions during the production, but sometimes processing, transport and trade are also included in the calculation.

**Table 1:** Examples of Carbon Footprints (CF) (kg CO_2eq_/kg milk) depending on the type of production.

Type of production/farming			References
Country	Conventional	Organic	
Germany	0.83	0.84	[18]
Germany	0.85	0.78	[14]
Sweden	0.90	0.94	[19]
Germany	0.94	0.88	[13]
The NL	0.97	1.13	[20]
Germany	0.98	0.92	[21]
Sweden	0.99	0.94	[22]
UK	1.06	1.23	[12]
Austria	1.20	1.00	[23]
UK	1.20	1.30	[24]
Germany	1.30	1.30	[25]
The NL	1.40	1.50	[26]
UK	1.6 (1.0–3.2)	1.3 (0.9–2.4)	[27]
**Without differentiation in conventional/organic**			
Germany	0.40 (40 kg milk/day)		[28]
(model calculation)	0.55 (20 kg milk/day)		[28]
	1.00 (10 kg milk/day)		[28]
Germany	0.65		[29]
New Zealand	0.65–0.75		[30]
Literature review	0.8–1.4 (on farm)		[8]
	0.9–1.8 (on farm + post farm emissions)		
New Zealand	0.86		[31]
Germany	0.98 (10,000)–1.35 (6,000 kg milk/year; see Table 3)		[32]
Sweden	1.00		[33]
Canada	1.00		[34]
UK	1.06		[12]
USA	1.09		[35]
EU-27	1.3 (1.0–2.3)		[7]
Ireland	1.3–1.5		[36]
Global	2.4 (1.3–7.5)		[4]

Still higher variations of CF are described for beef (see Table 2). The values are influenced by body weight gain, feeding, production system and system limits. There are many ways for CF calculation of the yield of growing animals such as body weight gain, hot standard carcass weight, empty body weight, meat, meat plus edible organs or edible protein. In dependence on the calculation basis, the authors found a high variation in CF for beef. The highest values are given for beef cows (Table 2). In general all the results indicate (e.g., [6,37,38]) that activities which are targeted at improvements in productivity and efficiency of resource use will result in a lower GHG emission or lower CF per unit of product.

**Table 2:** Examples for CF (kg CO_2eq_/kg carcass weight gain) of beef cattle depending on type of production.

Type of production/farming			References
Country	Conventional	Organic	
Germany	8.5	29.0 (beef cow)	[39]
Germany	8.7/10.1	10.2	[18]
Australia	9.9(grain finished)	12.0(grass finished)	[37]
Global	10	32–40	[24]
	(intensive–dairy beef)	(organic–suckler beef)	
Germany	13.3	11.4	[13]
Germany	15.2	17.5	[40]
UK	15.8	18.2	[12]
Ireland	23.6	20.2	[41]
Global	24.5	20.9	[42]
**Without differentiation in conventional/organic**			
Germany	5.6 (6,000)–14.6 (10,000 kg milk per cow per year, see Table 3)		[32]
Canada	5.9–10.4		[34]
Germany	7.0–23.0		[28]
Germany	8.4 (fattening of calves from dairy cows)		[14]
	16.8 (fattening of calves from beef cows)		
Sweden	10.1		[43]
Ireland	13.0 (11.3–15.6)		[41,44]
Global	15.6 (fattening of calves from dairy cows)		[4]
	20.2 (fattening of calves from beef cows)		
EU	16.9–19.9 (fattening of calves from dairy cows)		[45]
	27.3 (fattening of calves from beef cows)		
Japan	19.6		[46]
Japan	36.4 (beef cows, fattening bulls; 40% meat yield)		[47]

Apart from the factors mentioned above, the allocation of animal products (e.g., [15,26,32,43,48]) may be used whenever systems under study generate more than one saleable output (e.g., milk and meat) or various co-products. Such studies also influence the results of LCA (e.g., [32,37,48]). Mass-based and economic-based allocations were applied. For example Zehetmeier *et al.* [32,38] calculated CF of 1.35 and 0.98 kg CO_2eq_ per kg milk of cows producing 6,000 and 10,000 kg milk per year, respectively. In the case of lower milk yield, beef was produced by calves of dairy cows with a CF of 5.58 kg; in the case of higher milk yields, beef cows are needed to produce sufficient beef and the CF increased to 14.62 kg CO_2eq_ per kg beef. Under consideration of economic aspects (prices for milk and beef; economic allocation), the CF for milk decreased, those of beef increased. 

Under consideration of all aspects mentioned above, it is extremely difficult to compare results of LCA from different authors. This variability caused confusion between scientists, among policy makers and in the public. A methodical agreement generated by internationally recognized scientific panels with expertise across a range of disciplines and clear science based orientation (e.g., [9,16,24]) seems to be urgently necessary. 

Based on these, the objective of the present review is to characterize the most important influencing factors along the food chain for calculation of CF for food of animal origin. The next section deals with the whole food chain and its system boundaries followed by a critical assessment of the different kinds of animal yields or specific outputs of animal production under special consideration of edible protein and methods of their measurement/assessment.

## 2. Setting the Boundaries of a Production System and Further Influencing Factors Along the Food Chain

### 2.1. Emissions Along the Food Chain

The IPCC [2] recommended GHG factors for CO_2_ (1), CH_4_ (23) and N_2_O (296) to calculate CF for various processes. Recently there was some discussion about the factors and the IPCC [49] revised the global warming potential of methane (CH_4_) from 23 to 25 because of indirect effects of CH_4_ on ozone and stratospheric water vapour [9]. On the other hand, the Worldwatch Institute [50] suggests a Global Warming Potential for CH_4_ of 72. Methane is a very important gas for CF calculation, especially for food derived from ruminants. Between 50 and 80% of the total GHG emissions of food of ruminant origin are due to methane [37,38]. 

The N_2_O-factor is given with around 300 (296 [2]; 298 [49]). From a global view the agricultural N_2_O-emissions (from manure and soil; given in billion tonnes of CO_2eq_/y) are larger than calculated methane emissions (2.5 *versus* 2.15 billion t CO_2eq_/y, [6]). Analogue tendencies are reported from agriculture in the USA (222 million t CO_2eq_/y from N_2_O; 197 million t CO_2eq_/y from CH_4_; [51]). From the view of science and policy, further research is required to considering a time horizon of the GHG emissions [9,52].

In addition to GHG factors a possible exact measuring of climate relevant gases in all links along the food chain is an essential prerequisite to calculate CF.

**Figure 1:**
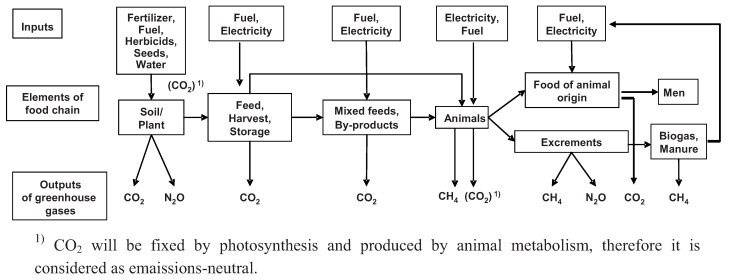
Substantial elements of the chain to produce food of animal origin, as well as selected inputs of resources and outputs of greenhouse gases (basic concept for system boundaries [17]).

There is general agreement that carbon dioxide emissions from livestock metabolism are not considered as a CO_2_-source in CF ([2,9], see Figure 1). CO_2_ has been fixed by photosynthesis in phytogenic biomass and the C consumed in feed and emitted as CO_2_ by animals is considered as equivalent or as emission neutral. On the other hand, CO_2_ from technical processes associated with animal husbandry should be considered in CF calculations (for details see [7,10,11]). 

Methane can be considered as an unavoidable by-product of anaerobic microbial fermentation, especially in the rumen of ruminants, but also in the hindgut of all species and during anaerobic manure management. Since energy losses via methane in the rumen are well known [53], animal nutritionists have been trying to reduce the gastrointestinal methane emission from ruminants and in the hindgut of further species for a long time. In 2005 around 90 million tonnes CH_4_ (about 1.9 billion t CO_2_eq/y) were emitted from gastrointestinal fermentation of ruminants [6]. These emissions are projected to grow by over 30% from 2000 to 2020 [6]. Enteric methane emissions (e.g., [54,55,56,57]), methods of measurements (e.g., [58,59,60,61]) and reduction potentials (e.g., [62,63,64,65]) should not be further considered here. Furthermore there are special issues of the journals “*Animal Feed Science and Technology*” 145 (2008), pp. 209–419 and 166–167 (2011), pp. 1–782 and in the “*Australian Journal of Experimental Agriculture*” 48, No. 1 and 2 (2008), dealing with the topics mentioned above.

Laughing gas (N_2_0) has the highest GHG potential from the most relevant GHG [2]. It is not directly excreted by animals; it depends on microbial conditions during manure management and in the soil. Lower N-excretion by animals [66,67,68] and improved N-management (e.g., storing of manure, adequate amounts of manure and fertilizer) may contribute to lower N-emissions from manure and soil (e.g., [69,70,71,72]). More representative results from N_2_O measurements (also for grazing animals) may substantially contribute to more reliable data for calculation of CF along the food chain. In some cases specific emissions were excluded from calculations of CF due to data uncertainty (e.g., N_2_O from leguminous pastures [37]). Such special situations should be mentioned in the study report to assess the results.

### 2.2. Setting the System Boundaries

Definition of system boundaries along the food chain (see Figure 1 and [24]) is the starting point for GHG measurements, for the calculation of CF of livestock products and for comparing results from various studies [4,10]. There are some open questions which need to be answered and it should be clearly defined whether they are or are not considered in the calculation, such as:

− Consideration of emissions from land use and land use change [1]
− Emissions from basic equipment (e.g., houses, machinery *etc.*; [38,73])
− Transport, processing, trade of products of animal origin [38]
− Emissions during preparing food from animal products in the kitchen/food processing
− Use of an allocation of various animal products (see above)

For example under Australian conditions Peters *et al.* [37] defined the system boundary in the case of beef for all on-site and upstream processes at the farm, feedlot, and whole processing plant, including transport between these sites. 

Table 3 shows exemplary CF for milk under consideration of various boundaries. A clear definition of the system boundaries and the comprehensibility are important prerequisites to follow the calculations and to make the results comparable (e.g., [37,41,44]). Scientists working in this field should come to agreements concerning system boundaries and GHG factors of climate relevant gases.

**Table 3:** Model calculation to demonstrate the effects of setting different boundaries for CF of milk (g CO_2eq_ per kg milk; 30 kg milk per day; diet on DM-base: 60% roughage, 40% concentrate; 4% milk fat, 3.4% protein; 305 days of lactation; 60 days dry period, 3 years lactation; 30 months calf and heifer period [38]).

System	System boundaries	CF (g CO_2eq_/kg milk)
1	Dairy cow emissions during lactation	280
2	1. + Emissions of feed production	430
3	2. + Dry period	500
4	3. + Heifer period	730
5	4. + Animal housing and milking	760
6	5. + Manure management	820
7	6. + Processing, transportation and trade of milk	1,100

## 3. Outcome of Animal Production

There is no essential need for food of animal origin for human beings, but the consumption of milk, eggs, meat and fish may substantially contribute to a more balanced and palatable human diet. Therefore one of the main goals of animal husbandry is the production of food of animal origin. Such food contributes substantially to meeting the human requirements in essential amino acids (e.g., [74,75,76]) because of the high content in essential amino acids (such as lysine, methionine and cysteine, threonine, leucine, *etc.*; see [77]). Furthermore, such food contains important minor nutrients like major and trace minerals (such as Ca, P, Cu, Fe, I, Se, Zn) and vitamins (e.g., A, E, some B-vitamins, especially B_12_) and has a considerable enjoyment value [78]. Human nutritionists [79,80] recommend that about one third of the daily protein requirement (0.66–1 g per kg body weight and day; [75,80,81]) should originate from protein of animal origin to guarantee a more balanced diet, especially for “risk groups” such as pregnant and lactating women, infants and children. Therefore, one endpoint, or the outcome of specific animal yields could be edible protein or essential amino acids and should be clearly defined. Otherwise there will be discrepancies in calculations and variations in the results between various working groups as shown in Table 1 and Table 2.

### 3.1. Milk and Eggs

Milk and eggs are clearly defined as food of animal origin. The yield can be measured as weight (kg, *etc.*) or on the basis of standardized products (e.g., standardized protein, fat, dry matter or energy). Therefore it is relatively easy to measure the animal yield for further calculations. But nevertheless, there is a certain range between CF for milk (see Table 1).

The composition of milk and eggs is well defined (see Table 6), but it may vary between various sources and depending on animal breed, feeding and other influencing factors. Therefore the analysis of milk and egg composition (protein; fat, lactose) may contribute to being more specific in measuring the animal yield incl. the energy yield. Milk and eggs may be used entirely as food (except small amounts of colostrum and egg shells; see Table 7).

### 3.2. Food from Slaughtered Animals

It is much more difficult to quantify and characterize the yield from the animal body after slaughtering and processing. The GHG balance per kilogram body weight gain can only be calculated on the farm level. Mialon *et al.* [82] carried out a feeding study with Blond d’Àquitaine bulls in the finishing phase (400–650 kg body weight) including various feeding systems and weight gains between 1,494 and 1,862 g/d and Doreau *et al.* [83] calculated a CF between 3.6 and 4.7 kg CO_2eq_ per kg body weight gain. Those values are similar to high daily weight gains as shown in Table 5, but much lower than for lower weight gains. Normally, the GHG emissions for the whole beef system include also emissions of cows, calves and heifers, needed to produce beef. They are much higher than in the system dairy cow—growing/fattening bulls for beef (see allocation).

Mostly the term “meat” is used, but it is not clearly described, what it means (real meat or meat plus bones). Peters *et al.* [37] introduced the term “hot standard carcass weight” (HSCW) as the weight at the exit gate of the meat processing plant. It varies between 50–62% of the live weight of the cattle to be slaughtered, but it may vary between 50% in the case of sheep and up to 80% for fattening turkeys (e.g., [12,37,83]).

In the case of animals for meat/fish production the following endpoints can be measured:

− Weight gain of the animal (per day or per growing period) during the whole life span
− Weight gain of animal without gastro-intestinal tract
− Empty body weight (or carcass weight; meat and bones; warm as HSCW or cold)
− Meat (empty body minus bones)
− Edible fraction (meat plus edible organs and tissues)
− Edible protein (edible fractions of the carcass multiplied with their specific protein content).

Therefore it is really difficult to find an adequate CF for meat or edible products from slaughtered animals. Various authors used different bases to calculate CF for products from slaughtered animals. Williams *et al.* [12] estimated the killing out percentages for beef and poultry with 55 and 70% and 72, 75 and 77% for pigs with live weights of 76, 87 and 109 kg, respectively. Lesschen *et al.* [7] used fixed values to calculate the carcass fraction from the final body weight of animals (e.g., 58% for beef; 75% for pork and 71% for poultry). Most authors used a fixed fraction of 0.9 for all animal species for conversion of carcass weight to edible “meat”. De Vries and de Boer [5] used calculation factors to determine the amount of edible product per kg live weight by 0.43; 0.53 and 0.56 for beef, pork and poultry. Table 4 shows potential outputs for growing/fattening cattle under consideration of various endpoints as mentioned above.

Calculation of CF may base on various outputs. For practical reasons carcass weight or weight gain (warm or cold) would be the most important endpoint to measure the yield of slaughtered animals because this weight is measurable in the abattoir [37] and can be used for further calculations. Based on the values derived from Table 4, CF is calculated for various endpoints under consideration of differences in feeding and greenhouse gas emissions and is shown in Table 5.

**Table 4:** Model calculation to show various endpoints for growing/fattening bulls (150-550 kg body weight; calculation based on data collected by [84]).

Gross weight gain (g/day)	Weight gain without content of intestinal tract (g/day)	Carcass weight (warm; % of weight gain)	Carcass weight gain (warm; g/day)	Meat gain (% of weight gain)	Meat gain (g/day)	Edible fraction gain ^1^ (g/day)	Edible protein (g/day; 19% protein in edible fraction)
500	438	50	250	40	200	250	48
1,000	900	53	530	44	440	490	93
1,500	1,385	56	840	48	720	770	146

^1^ Meat plus other edible tissues.

**Table 5:** Model calculations for CF of beef (150-550 kg body weight ^1^) depending on feeding, weight gain, methane- and N_2_O-emissions and N-excretion [28]

Weight gain (g/day)	Feed intake (kg DM/ (animal x day)	Portion concentrate (% of DM-intake) ^1,2^	Methane emissions (g/kg DM)	N-excretion (g/day)	N_2_O-synthesis (% of N-excretion)	Carbon footprints (kg CO_2eq_/kg)			
						Weight gain	Empty carcass weight gain	Edible fraction gain	Edible Protein
500 (Pasture, no concentrate)	6.5	0	26	110	2	11.5	23.0	28.0	110
1,000 (Indoor, grass silage, some concentrate)	7.0	15	24	130	1	5.5	11.0	13.8	55
1,500 (Indoor, corn silage, concentrate)	7.5	30	22	150	0.5	3.5	7.0	9.0	35

^1^ Production of calf up to 150 kg BW is not considered; ^2^ CO_2_-Emission: 120 g/kg roughage-DM; 220 g/kg concentrate-DM.

### 3.3. Edible Protein as Most Important Objective of Animal Husbandry

The production of protein of animal origin is one of the most important goals of animal husbandry [5]. On the other hand, the efficiency and the emissions of animal products can be also compared on the basis of edible protein. The N or protein content of various foods of animal origin may vary from values used for calculations in Table 6 (data by [84] on the basis of own studies). Our data agrees with values used by Lesschen *et al.* [7], and it does not substantially disagree with values from human food tables (see Table 6). De Vries and de Boer [5] used for their calculations 190 g protein/kg edible beef, pork and poultry meat; 30 g per kg milk products and 130 g per kg eggs.

Considering various influencing factors such as animal yields, feeding, edible fractions and protein content in the edible fractions, the yield of edible protein per day and per kg body weight of animals is given in Table 7. 

**Table 6:** Published data regarding the protein content of some edible animal products (in g per kg edible product).

Product	References						
	[7] ^1^		[77]		[84]	[85]	[86,87,88,89]
Cows milk	34.4		33.3 (30.8-37.0)		32	34	34
Beef	206		220 ^2^ (206-227)		190	206-212	170-200
Pork	156		220 ^2^ (195-240)		150	183-216	157 (129-178)
Broiler	206		199		200	182-242	n.d.
Eggs	119		125		120	125	121 (110-124)

^1^ N-content × 6.25; ^2^ Muscles only; n.d.: no data.

The feeding may influence CF of food of animal origin. In the case of ruminants, higher amounts of concentrate are required for higher animal yields. The proportion of by-products [90,91] used in animal feeding does not only have nutritional implications, but it also affects the results of calculations on land use [92]. There are large differences in protein yield per animal per day or per kg body weight and day depending on animal species and category as well as their performances and the fractions considered as edible (see Table 7).

**Table 7:** Influence of animal species, categories and performances on yield of edible protein [84].

Protein source (Body weight)	Performance per day	Dry matter intake (kg per day)	Roughage to concentrate ratio (on DM base, %)	Edible fraction (% of product or body mass)	Protein in edible fraction (g per kg fresh matter)	Edible protein (g per day)	Edible protein (g per kg body weight and day)
Dairy cow (650 kg)	10 kg milk	12	90/10	95	34	323	0.5
	20 kg milk	16	75/25			646	1.0
	40 kg milk	25	50/50			1292	2.0
Dairy goat (60 kg)	2 kg milk	2	80/20	95	36	68	1.1
	5 kg milk	2.5	50/50			170	2.8
Beef cattle (350 kg)	500 g ^1^	6.5	95/5	50	190	48	0.14
	1,000 g ^1^	7.0	85/15			95	0.27
	1,500 g ^1^	7.5	70/30			143	0.41
Growing/fattening pig (80 kg)	500 g ^1^	1.8	20/80	60	150	45	0.56
	700 g ^1^	2	10/90			63	0.8
	1,000 g ^1^	2.2	0/100			81	1.0
Broiler (1.5 kg)	40 g ^1^	0.07	10/90	60	200	4.8	3.2
	60 g ^1^	0.08	0/100			7.2	4.8
Laying hen (1.8 kg)	50% ^2^	0.10	20/80	95	120	3.4	1.9
	70% ^2^	0.11	10/90			4.8	2.7
	90% ^2^	0.12	0/100			6.2	3.4

^1^ Daily weight gain, ^2^ Laying performance.

Table 7 shows the highest protein yields per kg body weight for growing broilers as well as for laying and lactating animals and the lowest values for growing/fattening ruminants. Based on those values, emissions per kg edible protein are given in Table 8. Higher proportions of edible fractions or higher protein content (e.g., 50 g protein per kg camel milk, [77]) as shown in Table 6 and Table 7 may increase the protein yield and reduce the CF per unit of product.

Apart from protein food of animal origin also contains fat and some carbohydrates which contribute to human nutrition and which may replace energy of plant origin in human diets. 

At high levels of performance there are remarkable differences in CO_2_ emissions due to human consumption of 1 g protein from food of animal origin (eggs and meat from broiler < pork < milk < beef, see Table 7). But here it has to be emphasized that this protein intake is accompanied—willingly or not—by an energy intake from the protein itself, but also from further nutrients like lactose and fat in milk or from fat in meat or eggs. Therefore, it should be avoided to attribute the CO_2_ burden to the protein fraction (“edible protein”) exclusively. To prevent that this fact is neglected, there are different alternatives:

In a first simple method, the CO_2_ emission due to 1 kg edible protein could be used as CO_2_ burden of consumed energy (for example: 1 kg edible protein of eggs corresponds to about 8 kg egg corresponding to 51.6 MJ energy (calculated by [77]); these combined intakes are related to 3 kg CO_2_).

One alternative could be a “nutritional allocation” (as described before for economic allocation), meaning that the CO_2_ emissions are attributed to different functions of the food (source of protein/source of energy/source of further essential nutrients). 

**Table 8:** Influence of animal species, categories and performances on emissions (per kg edible protein, own calculations).

Protein source (Body weight)	Performance per animal per day	N-excretion (% of intake)	Methane emission (g per day) ^3^	Emissions in kg per kg protein			
				P	N	CH_4_^3^	CO_2eq_
Dairy cow (650 kg)	10 kg milk	75	310	0.10	0.65	1.0	30
	20 kg milk	70	380	0.06	0.44	0.6	16
	40 kg milk	65	520	0.04	0.24	0.4	12
Dairy goat (60 kg)	2 kg milk	75	50	0.08	0.5	0.8	20
	5 kg milk	65	60	0.04	0.2	0.4	10
Beef cattle (350 kg)	500 g ^1^	90	170	0.30	2.3	3.5	110
	1,000 g ^1^	84	175	0.18	1.3	1.7	55
	1,500 g ^1^	80	180	0.14	1.0	1.2	35
Growing/fattening pig (80 kg)	500 g ^1^	85	5	0.20	1.0	0.12	16
	700 g ^1^	80	5	0.12	0.7	0.08	12
	900 g ^1^	75	5	0.09	0.55	0.05	10
Broilers (1.5 kg)	40 g ^1^	70	Traces	0.04	0.35	0.01	4
	60 g ^1^	60		0.03	0.25	0.01	3
Laying hen (1.8 kg)	50% ^2^	80	Traces	0.12	0.6	0.03	7
	70% ^2^	65		0.07	0.4	0.02	5
	90% ^2^	55		0.05	0.3	0.02	3

^1^ Daily weight gain ^2^ Laying performance ^3^ CH_4_-emission varies with composition of diet.

In a first simple step it is recommended to diminish the CO_2_ emission per 1 kg edible protein (Table 8) by the CO_2_ amounts that would occur at an identical energy intake from food of plants (energy from carbohydrates and fat). It means that an intake of 1 kg protein from eggs (corresponds to 8 kg eggs; see Table 7; and corresponding to 51.6 MJ energy) saves high amounts of other food (and their CO_2_ burden). A more sophisticated way of an “allocation” within the foods could be to differentiate between “protein derived energy” and “non-protein derived energy”. In milk and eggs more than 50% of the total energy content is related to the non-protein-fraction (lactose/fat), therefore, it is questionable whether the entire CO_2_ emission should be attributed only to the protein intake. Due to the very low CO_2_ emission caused by energy intake of carbohydrates and fat from plants/seeds [12,15,17] it would avoid/save high amounts of CO_2_ emissions, if the production of food of animal origin focussed on “edible protein” and not on energy of non-proteinaceous fractions.

Furthermore animal products are not only used as food or respectively, as protein/amino acids, and energy sources; they also offer some other important side-products such as skins or hides, fish meal or meat and bone meal, *etc.* A kind of combined “nutritional/further purposes allocation” may contribute to a more scientific assessment of CF for nutrient and energy supply as well as further uses.

Advantages and weaknesses of endpoints (outputs) of various types of animal production are summarized in Table 9. All endpoints are characterized by some advantages and disadvantages. From nutritional and scientific points of view the edible protein seems to be the most favourable measurement, but its measurement is not easy and requires some analytical work (see Table 9). Land requirements (e.g., arable land and/or grassland) as well types and intensities of food production may be calculated on the basis of various protein sources for human nutrition. Such calculations can contribute for better understanding of various conflicting aims in the field of food production, human nutrition, use of unlimited and limited resources and resource efficiency, emissions and further points in public discussion.

**Table 9:** Advantages and disadvantages of various outputs/endpoints of animal yields.

Animal yields	Advantages	Disadvantages
Milk, Eggs	Easily measurable, almost complete edible	Variation in protein, fat and energy yield, analyses may be useful
Body weight gain	Easily measurable	High portion of non edible fractions in the gains
Carcass weight	Easily measurable	Contains still fractions which are not edible (e.g., bones)
Meat, edible fraction	Completely edible	Categorization and separation not easy
Edible protein	Most important objective of animal production; comparison of various methods and sources to produce protein of animal origin	Categorization of various fractions as edible and difficulties to measure; additional analytical work; variation in N/protein content

## 4. Conclusions

Ranking of food of animal origin on the basis of CF may be indicative for some products, but may also lead to wrong conclusions because of incompleteness of measuring animal yields and data bases, system boundaries and other weaknesses. The data bases for GHG emissions should be improved and the animal yields should be made comparable. Edible protein (or rather, essential amino acids and some minor nutrients) of animal origin, being the most important objective of animal husbandry, is proposed as a standard to compare various types and intensities of animal husbandry. Furthermore we have to look at the whole food chain (see Figure 1) in order to decide whether a practice is sustainable or not in the long term (e.g., [93,94]). In order to do this, however, further research is needed for more reliable and resilient data.
